# Inhibition of stearoyl CoA desaturase‐1 activity suppresses tumour progression and improves prognosis in human bladder cancer

**DOI:** 10.1111/jcmm.14114

**Published:** 2018-12-27

**Authors:** Chiyuan Piao, Xiaolu Cui, Bo Zhan, Jun Li, Zeliang Li, Zhenhua Li, Xiankui Liu, Jianbin Bi, Zhe Zhang, Chuize Kong

**Affiliations:** ^1^ Department of Urology The First Hospital of China Medical University Shenyang Liaoning P.R. China

**Keywords:** cancer stem cells, cell proliferation, stearoyl CoA desaturase‐1, urinary bladder neoplasm

## Abstract

Urinary bladder neoplasm is one of the most common cancers worldwide. Cancer stem cells (CSCs) have been proven to be an important cause of cancer progression and poor prognosis. In the present study, we established bladder CSCs and identified the crucial differentially expressed genes (DEGs) between these cells and parental bladder cancer cells. Analyses of bioinformatics data and clinical samples from local hospitals showed that stearoyl CoA desaturase‐1 (SCD) was the key factor among the DEGs. A significant correlation between SCD gene expression and poor prognosis among patients with bladder cancer was observed in our data. Loss‐of‐function experiments further revealed that the SCD inhibitor A939572 and SCD gene interference reduced cell proliferation and invasion. The above data suggest that SCD may serve as a novel marker for the prediction of tumour progression and poor prognosis in patients with bladder cancer.

## 1 INTRODUCTION

In western countries, bladder urothelial cell cancer is the fourth most common malignancy in men. It has been estimated that there are more than 700 000 bladder carcinoma survivors living in the US.^1^ The Surveillance, Epidemiology and End Results (SEER) program of the National Cancer Institute showed that the incidence of bladder cancer is approximately 4 times higher in men than in women. More than 70% of patients who have bladder cancer are initially diagnosed with non‐muscle‐invasive disease. Based on SEER data, combined with all stages, 5‐year survival rate is over 70%. Survival goes down to 70% at 10 years and to 65% at 15 years after the first diagnosis. In China, bladder carcinoma is the fifth most common malignant tumour in men. The overall mortality of bladder carcinoma is 25.1%.^2^

A thorough epidemiological article presented an overview of recent patterns and trends in bladder cancer incidence and mortality in different countries and analysed the influence of known aetiological factors. The authors identified several implications of the disease and its behaviour and revealed the efficacy of current efforts and measures.^3^ Up to 70% of patients with non‐muscle‐invasive bladder cancer (NMIBC) could suffer tumour recurrence, and approximately 10%‐15% of these patients progress to muscle‐invasive bladder cancer (MIBC).^4^ Therefore, accurate prediction of tumour recurrence and progression is important for determining the appropriate therapy and disease follow‐up.

Cancer cells are heterogeneous and are assumed to include cancer stem cells (CSCs). The experimental evidence of CSCs was first described in haematology.^5^, ^6^ According to the consensus definition of CSCs,^7^ these cells have the capacity for self‐renewal and the ability to generate the heterogeneous lineages of cancer cells that compose the tumour.

The remarkable pathological differences between NMIBC and MIBC lead to substantial differences in disease progression and outcome. Compared with NMIBC, MIBC consists of a wider variety of cancer cells that range from differentiated to undifferentiated. Urothelial stem cells localize in the basal cell layer and generate all different types of urothelial cells.^8^ When the urothelium is partially injured, urothelial stem cells are capable of regeneration and compensation for the damaged tissue. However, the regenerative trait of stem cells is strictly co‐ordinated in the normal urothelial lineage, thereby preventing tumour formation. Bladder CSCs (BCSCs) were first identified by sorting with markers of normal basal cells.^9^ Many studies have reported new methods for identifying BCSCs, and the results progressively support the existence of BCSCs.^10^ Nevertheless, the origin of BCSCs and their mechanism of differentiation are mostly undiscovered.^11^

In the present study, we suggested that BCSCs are responsible for the poor prognosis of patients with bladder cancer. Therefore, we selected a target gene, stearoyl CoA desaturase‐1 (SCD), among the differentially expressed genes (DEGs) between BCSCs and their parental bladder cancer cells. Recently, the critical participation of SCD that converts saturated fatty acids (SFAs) into monounsaturated fatty acids (MUFAs), in the mechanisms of proliferation and survival in mammalian cells and the implications of these fatty acids for the biological effects associated with malignancy have been actively researched. To verify the potential biological functions of SCD in bladder cancer, we performed several cancer‐related assays.

## 2 MATERIALS AND METHODS

### 2.1 Cell culture and reagents

The human bladder cancer cell lines 5637, T24, UMUC3 and J82 were cultured in RPMI‐1640 medium, RT4 cells were cultured in McCoy’s 5A medium and TCCSUP cells were cultured in Dulbecco’s modified Eagle’s medium (DMEM). All culture media were supplemented with 10% foetal bovine serum. All cancer cell lines were obtained from the Type Culture Collection of the Chinese Academy of Sciences (Shanghai, China) and cultured under humidified air containing 5% CO_2_ at 37°C. When the cells reached over 80% confluence, they were washed with 1× PBS and trypsinized at 37°C for a specified number of minutes for cell passage cultivation.

A939572 was purchased from MedChem Express. The catalog number is HY‐50709.

### 2.2 Isolation and propagation of BCSCs

Monolayers of 5637 and T24 BCSCs were dissociated in trypsin (0.25%)/EDTA, plated in ultralow attachment flasks (Corning) at a density of 10 000 cells/mL and grown in serum‐free medium (DMEM/F12; 1:1 mixture) containing B27 (Invitrogen) and supplemented with recombinant epidermal growth factor (EGF) at 20 ng/mL (Invitrogen) and recombinant bFGF at 10 ng/mL (Invitrogen). Each generation of CSCs was cultured for 7‐10 days and the sphere cells were subcultured using trypsin and resuspended in serum‐free medium described above. The third generation spheres were used for identification of stemness and microarray analysis.

### 2.3 Microarray analysis

Samples of total RNA from the parental 5637/T24 cell line and 5637/T24 sphere cells were analysed on an Affymetrix HTA 2.0 Array. The arrays were scanned by an Affymetrix GeneChip^®^ Scanner 3000 (Cat# 00‐00213; Affymetrix, Santa Clara, CA, USA). Command Console Software (Affymetrix) was used with the default settings to control the scanner and summarize probe cell intensity data (CEL file generation). Then, the raw data were normalized by Expression Console.

### 2.4 Data pre‐processing and DEG screening

The probe‐level data in CEL files were converted into an expression value matrix by GCBI online platform analysis at the following link: http://www.gcbi.com.cn. Robust Multichip Average, including the background‐correction, normalization and summary features, was used to compute the expression value. Quality control of the gene expression data was performed using a gene‐specific probe. The median normalized unscaled standard error value of each chip was applied to evaluate the feasibility of the design and the reliability of the analysis results. We further assessed the change rule of every probe set in the experiment by computing the relative log expression (RLE). The unified standard criterion for every sample was as follows: (1‐0.2) <median <(1 + 0.2) and (−0.25) <median {RLE} <(0.25). Chips that deviated far from this criterion were rejected.

To identify DEGs between the parental 5637/T24 bladder cancer cells and 5637/T24 CSCs, only probe signals with *P*‐values <0.01, *q*‐values <0.05 and absolute values of fold change (FC) >3 were considered to be significantly differentially expressed.

### 2.5 Bioinformatics analyses

To assess the survival curve and expression levels of SCD mRNA in the normal bladder vs bladder cancer, the ONCOMINE database (https://www.oncomine.org) and The Cancer Genome Atlas (TCGA) were interrogated. TCGA platform analysis is available at the following link: http://gepia.cancer-pku.cn.

### 2.6 Gene expression analysis by quantitative RT‐PCR

Total RNA was isolated from whole bladder cancer samples or bladder cancer cells using TRIzol reagent, treated with DNase I, and then used for cDNA synthesis with PrimeScript RT Master Mix. Quantitative RT‐PCR analyses were performed using LightCycler with SYBR Premix Ex Taq. Glyceraldehyde‐3‐phosphate dehydrogenase (GAPDH) was used as the internal reference.

### 2.7 Immunohistochemistry

Paraffin‐embedded tissue blocks were cut in a certain orientation at a thickness of 4 μm to obtain serial sections of the normal bladder tissue or bladder cancer. The slices were mounted on glass slides. Immunostaining was performed using the avidin‐biotin‐peroxidase complex method (Ultrasensitive™; MaiXin, Fuzhou, China). The sections were deparaffinized in xylene, rehydrated with graded alcohol (100%, 90%, 80%, 70%, 60% and 50%) and then boiled in 0.01 mol/L citrate buffer (pH 6.0) for 2 minutes in an autoclave. Peroxidase inhibitor was applied to block endogenous peroxide activity for 30 minutes, and the sections were incubated with normal goat serum to reduce non‐specific binding for 30 minutes. The tissue sections were incubated with an anti‐SCD mouse monoclonal antibody (1:30 dilution, Santa Cruz). Antibody staining was performed at 4°C overnight. Biotinylated goat anti‐mouse serum IgG was used as a secondary antibody. After washing, the sections were incubated with streptavidin‐biotin conjugated to horseradish peroxidase, and the immunoreaction was visualized using diaminobenzidine as a chromogen. As a control, incubation without the primary antibody or with non‐specific serum was also performed. Nuclear staining was carried out by treating the slides with hemalum for 2 minutes, followed by a 10‐minute incubation in running water to induce the colour reaction. Finally, the stained slices were dehydrated and mounted.

### 2.8 Western blotting

Whole‐cell lysates were extracted by using radioimmunoprecipitation assay buffer containing protease and phosphatase inhibitors. Protein fractions were separated by 10% SDS‐PAGE (140 V). The resolved proteins were transferred (350 mA) to PVDF membranes (0.2 μm) using a mini‐transblot apparatus (Bio‐Rad). The membranes were blocked with 5% non‐fat milk at room temperature for 2 hours, incubated with the primary antibody at 4°C overnight and then incubated for 45 minutes with the appropriate secondary antibody. Proteins were finally detected with Luminata substrate. Quantification of the immunoblots was performed using ImageJ software. All optical density values from immunoblots were normalized to the density values acquired for GAPDH.

### 2.9 Cell proliferation assay

The effect of A939572 on cell proliferation was assessed by counting viable cells using a colorimetric assay in the Cell Counting Kit‐8. Cells were seeded in 96‐well plates at 2000 cells per well for 24 hours, and A939572 was then added at concentrations of 0, 3.2 and 25.6 µg/mL for 24 hours. The absorbance at 450 nm was measured by an absorbance reader (Bio‐Rad).

### 2.10 5‐ethynyl‐2′‐deoxyuridine assay

Flow cytometry was performed with a FACSCalibur Flow Cytometer equipped with a 488 nm laser. UMUC3 and RT4 cells were transfected with siRNA or pre‐treated with A939572 at concentrations of 0, 6.4 or 12.8 µg/mL in 6‐well plates. Seventy‐two hours after transfection or 24 hours after treatment, 5‐ethynyl‐2′‐deoxyuridine (EdU) (50 µmol/L) (Cell‐Light™ EdU Apollo®488 In Vitro Flow Cytometry Kit, Guangzhou RiboBio, China) was added, and the cells were cultured for an additional 4 hours. The cells were then stained according to the following protocol. The EdU medium mixture was discarded, and 4% paraformaldehyde was added to fix the cells at room temperature for 15 minutes. The cells were washed with glycine (2 mg/mL) for 5 minutes, 0.5% Trion X‐100 was added for 10 minutes and the cells were washed two times with PBS. Then, click reaction buffer (Tris‐HCl, pH 8.5, 100 mmol/L; CuSO_4_, 1 mmol/L; Apollo 488 fluorescent azide, 100 µmol/L; and ascorbic acid, 100 mmol/L) was added for 10 minutes while protecting the samples from light. The cells were washed twice with 0.5% Triton X‐100, and 150 µL of PBS was added to resuspend the cells. Images were collected and analysed using BD CellQuest Pro software.

### 2.11 Sphere formation

For the sphere‐forming assay, 2000 viable cells per well were plated in 6‐well ultralow attachment flasks (Corning) and grown in serum‐free medium (DMEM/F12; 1:1 mixture) supplemented with EGF (20 ng/mL), B27 (1:50) and bFGF (20 ng/mL). The spheres were imaged after 10‐14 days.

### 2.12 Colony formation

Single cells were plated in a 6‐well plate at a density of 1000 cells per well in suitable medium. The cloning efficiency was calculated by ImageJ software.

### 2.13 Cell cycle analysis

Flow cytometry was performed with a FACSCalibur Flow Cytometer. All pre‐treated cells were harvested, washed twice with PBS and fixed with 5 mL of 70% ethanol at 4°C overnight. Then, the cells were washed twice in PBS and resuspended in 500 µL of PI/RNase Staining Buffer Solution (BD Pharmingen) in the dark at room temperature. Finally, cell cycle images were collected and analysed by using BD CellQuest Pro software and ModFit LT software.

### 2.14 Measurement of apoptosis by flow cytometry

Apoptosis was analysed with an FITC Annexin V Apoptosis Detection Kit (BD Pharmingen). After treatment, the cells were gently trypsinized and washed with serum‐containing medium. The cells were collected by centrifugation, washed with PBS and resuspended in 1X Binding Buffer. The cells were then stained with annexin V‐FITC and PI at room temperature for 5 minutes in the dark, according to the manufacturer’s instructions, and analysed in a FACSCalibur Flow Cytometer.

### 2.15 Cell invasion assays

Invasion assays were performed using BD 24‐well Boyden chambers. A total of 2 × 10^5^ cells were added to the top insert, and 500 µL of 10% FBS‐containing medium was added to the bottom chamber. After 24 hours of incubation, the cells that invaded the lower surface of the membrane were fixed, stained with 0.4% crystal violet and counted.

### 2.16 Cell migration assay

Cell migration abilities were measured in modified Boyden chambers consisting of Transwell membrane filter inserts. For all types of cells, serum‐starved cells (2 × 10^5^) suspended in 200 µL of suitable medium containing 5% FBS were cultured in each Transwell chamber and allowed to migrate towards the underside of the membrane for 24 hours. The lower chamber contained medium with 20% FBS. Cells that had not penetrated the filter were wiped away by a cotton swab, and the cells on the lower surface of the filter were stained with 0.4% crystal violet. The number of migrated cells was counted under a light microscope. Migration efficiency was calculated by ImageJ software.

### 2.17 In vivo tumourigenesis analysis

Cancer stem cells and their parental cells were subcutaneously injected into 4‐week‐old female nude mice in different amounts (10^3^, 10^4^, 10^5^, 10^6^ and 10^7^ cells). Tumour growth was observed every 3‐5 days. After 5‐6 weeks of observation, the injected nude mice were killed, and the tumourigenic ability between two groups at different cell amounts was compared.

### 2.18 Statistical analysis

Experimental data are presented as the mean ± SD from at least three independent experiments performed in triplicate; differences between groups were analysed by Student's *t* test. Differences between groups in clinical data were evaluated by Mann‐Whitney test or Dunn’s multiple comparisons test. Survival status was analysed by Kaplan‐Meier/Logrank methods. Statistical analysis was performed using GraphPad Prism version 7.0 software.

## 3 RESULTS

### 3.1 DEGs between BCSCs and common bladder cancer cell lines

**Figure 1:**
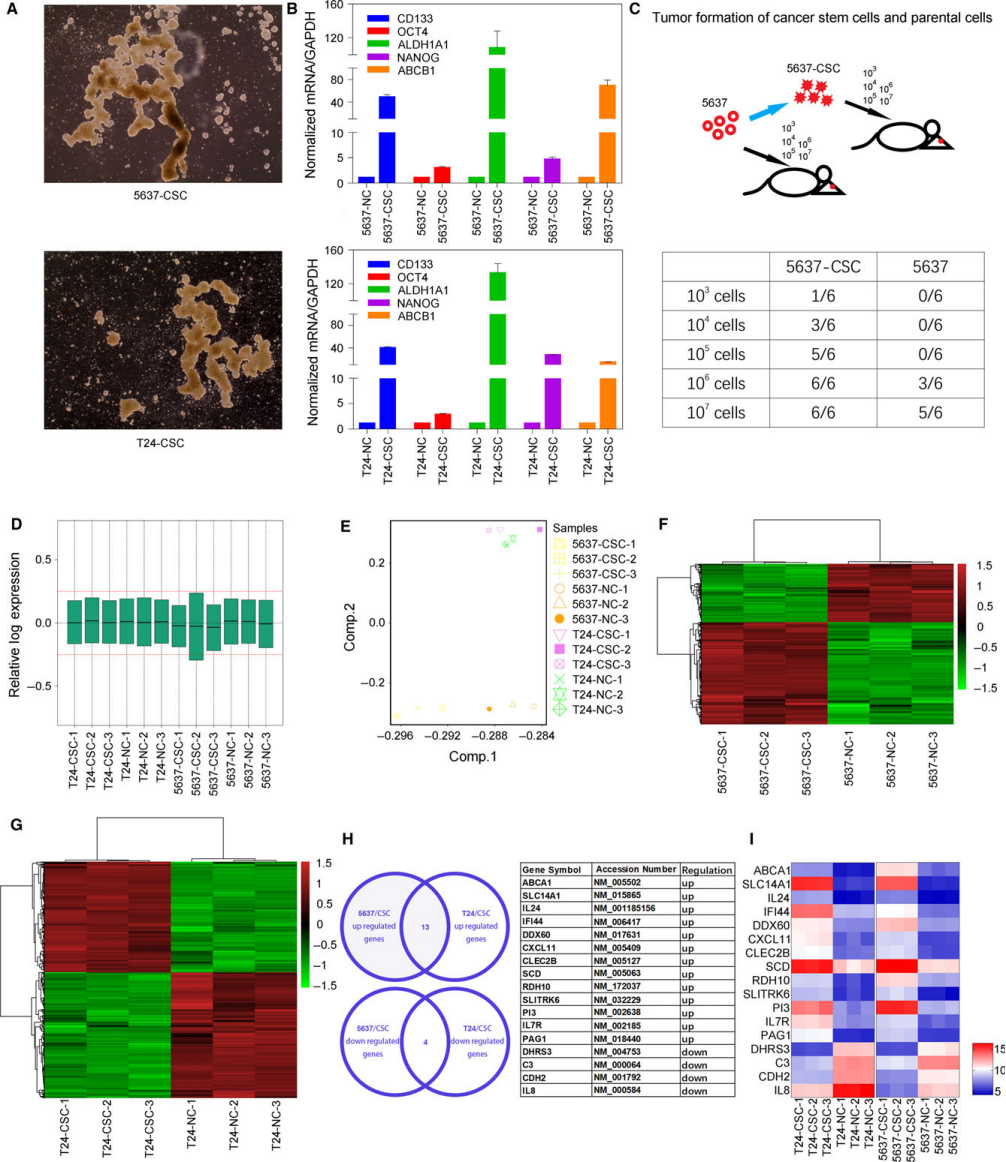
Thirteen up‐regulated genes and four down‐regulated genes were identified by analysing DEGs between BCSCs and common bladder cancer cell lines. A, The third generation spheres formed by 5637 and T24 cell lines. B, The mRNA expression levels of five stemness‐related regulators (CD133, OCT4, NANOG, ABCB1, ALDH1A1) are extremely up‐regulated in 5637 and T24 cancer stem cells compared to their parental cells. C, In vivo tumourigenesis analysis, 5637‐derived cancer stem cells or parental 5637 cells were subcutaneously injected into nude mice in varying amounts (10^3^, 10^4^, 10^5^, 10^6^ and 10^7^ cells). The tumourigenic ability of 5637 cancer stem cells is extremely enhanced. D, The quality control of the microarray assay. E, The unified standard criterion of the microarray assay. F, Heatmap of the altered gene expression profiles in 5637 CSCs and parental 5637 cells. G, Heatmap of the altered gene expression profiles in T24 CSCs and parental T24 cells. H, Thirteen up‐regulated genes and four down‐regulated genes were identified by intersecting up‐regulated DEGs or down‐regulated DEGs using GCBI (left panel). Gene symbols and accession numbers of selected genes were listed (right panel). I, Heatmap of the altered gene expression profiles of 17 selected genes based on T24 and 5637 related microarray assay

Using the human bladder cancer cell lines 5637 and T24, we isolated BCSCs by culturing 5637 or T24 cells in serum‐free DMEM/F12 (1:1) containing B27, recombinant EGF at 20 ng/mL and recombinant bFGF at 10 ng/mL. We cultured each generation of CSCs for 7‐10 days and the sphere cells were subcultured using trypsin and resuspended in serum‐free medium, then we used the third‐generation spheres for microarray analysis (Figure 1A). The total isolation and propagation time were about 30 days. Before using the CSCs for microarray assay, we examined the expression of several regulators of stemness and self‐renewal activity by qRT‐PCR, including CD133, OCT4, NANOG, ABCB1 and ALDH1A1. The mRNA expression levels of all five stemness factors are extremely up‐regulated in 5637 and T24 CSCs compared to their parental cells (Figure 1B). More importantly, tumour formation analysis was performed in nude mice by using 5637‐derived CSCs and their parental 5637 cells (T24 has no tumourigenic ability in nude mice). 5637 CSCs and their parental cells were subcutaneously injected into 4‐week‐old nude mice in varying amounts (10^3^, 10^4^, 10^5^, 10^6^ and 10^7^ cells). After 5‐6 weeks, we compared the differences in tumourigenic ability between two groups at different concentrations in nude mice. The results showed that compared with the parental cancer cells, the tumourigenic ability of cancer stem cells is significantly enhanced (Figure 1C). Next, we analysed the parental 5637/T24 cell line and 5637/T24 CSCs on an Affymetrix HTA 2.0 Array. Based on the quality control (Figure 1D) and the unified standard criterion (Figure 1E), we identified DEGs between the parental 5637 cells and 5637 CSCs (Figure 1F) and between the parental T24 cells and T24 CSCs (Figure 1G). Furthermore, to identify DEGs that were present in both DEG datasets, as shown, we intersected up‐regulated DEGs or down‐regulated DEGs using GCBI at the following link: http://www.gcbi.com.cn. Thirteen up‐regulated genes and four down‐regulated genes were identified, as displayed in the chart (Figure 1H). The heatmap shows the relative expression of each gene (Figure 1I).

### 3.2 High SCD mRNA and protein levels are associated with poor prognosis in patients with bladder cancer

**Figure 2:**
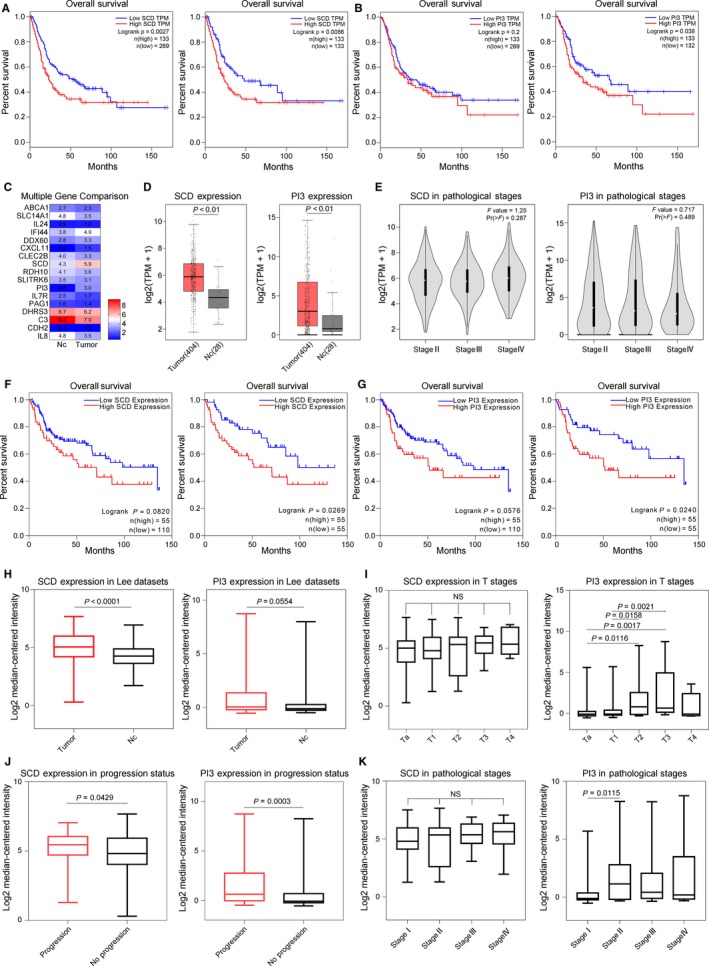
High SCD mRNA and protein levels are associated with poor prognosis in patients with bladder cancer. A, Using the TCGA platform at http://gepia.cancer-pku.cn, as shown by the Kaplan‐Meier curve, patients with high SCD mRNA (red curve) expression displayed a significantly lower overall survival than patients with low SCD mRNA (blue curve) expression in two different group cut‐off programs. B, Patients with high PI3 mRNA (red curve) expression displayed a significantly lower overall survival than patients with low PI3 mRNA (blue curve) expression in only one group cut‐off program. C, Heatmap of relative gene expression levels of 17 selected genes between non‐tumour tissue (n = 28) and cancer tissue (n = 404) based on the TCGA datasets. D, SCD (left boxplot) or PI3 (right boxplot) expression was up‐regulated in cancer tissue compared to that in non‐tumour tissue. E, SCD (left) or PI3 (right) expression have no correlation with pathological stage. F, Using ONCOMINE tool, patients with high SCD mRNA (red curve) expression displayed a significantly lower overall survival than patients with low SCD mRNA (blue curve) expression in one group cut‐off programs. G, Patients with high PI3 mRNA (red curve) expression displayed a significantly lower overall survival than patients with low PI3 mRNA (blue curve) expression in only one group cut‐off program. H, Unlike SCD (left boxplot), there was no significant difference in PI3 (right boxplot) gene expression between tumour (n = 188) and non‐tumour tissues (n = 68). I, PI3 (right) mRNA levels increased progressively from Ta to T3; there was no significant correlation between SCD (left) mRNA levels and T stage. J, SCD and PI3 were both associated with tumour progression. K, PI3 (right) mRNA increased as the pathological stage progressed from stage I to stage II; no significant correlation was shown between SCD (left) mRNA levels and pathological stage

To better clarify the possible associations between these 17 genes and patient survival status or gene expression in bladder cancer compared to normal bladder mucosal tissue, we first performed bioinformatics analyses using the TCGA platform at the following link: http://gepia.cancer-pku.cn. To improve the accuracy of the relationships between these 17 genes and survival status, two different group cut‐off programs were used. As shown by the Kaplan‐Meier curve, patients with high SCD mRNA expression displayed a significantly lower overall survival than patients with low SCD mRNA expression in both programs (Figure 2A).

High PI3 expression was found to be significantly correlated with worse patient survival in one group cut‐off program (Figure 2B). As shown in Figure 2D, SCD or PI3 expression was significantly up‐regulated in cancer tissue compared to that in non‐tumour tissue. There was no significant correlation between SCD or PI3 gene expression and pathological stage (Figure 2E). To confirm these results, we then used the ONCOMINE database (https://www.oncomine.org) to analyse survival status and differential gene expression. There are a total of nine bladder‐related datasets, including TCGA bladder data. The Lee bladder dataset is considered because it contains gene expression levels for both SCD and PI3, survival status, follow‐up time, TNM stage and progression status for patients with bladder cancer. Database analysis revealed similar results for survival conditions (Figure 2F,G). However, unlike SCD, there was no significant difference in PI3 gene expression between tumour and non‐tumour tissues (Figure 2H). We next analysed the relationship between SCD/PI3 levels and tumour progression status. As shown in Figure 2J, SCD/PI3 are both associated with tumour progression. The data suggested that PI3 mRNA levels progressively increased as the T stage progressed from Ta to T3 (Figure 2I) and as the pathological stage progressed from stage I to stage II (Figure 2K). In contrast, there was no significant correlation between SCD gene expression and T stage or pathological stage (Figure 2I,K). To sum up, as revealed in both TCGA and Lee bladder dataset, compared to PI3 related information, SCD showed more direct overall survival data and normal/tumour tissue differential expression data. Besides, the abundance of SCD is much more higher than PI3 in bladder cancer (Figure 2C,D,H). Therefore, SCD is a better target among 17 selected genes.

**Figure 3:**
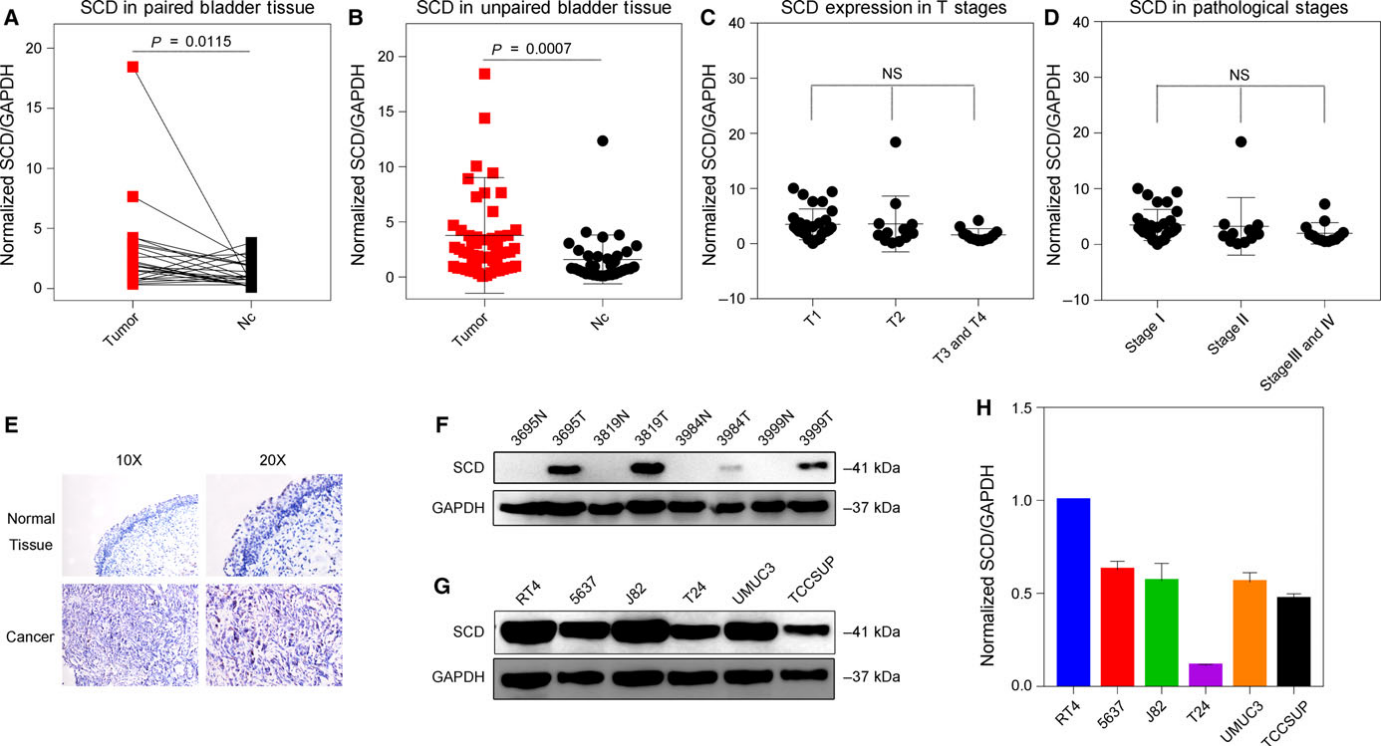
SCD expression was up‐regulated in cancer tissue compared to non‐tumour tissue in local clinical tissue specimens. A, SCD gene expression was significantly up‐regulated in tumour tissues compared to adjacent bladder mucosal tissues (n = 25 pairs). B, SCD gene expression was up‐regulated in tumour tissues (n = 57) compared to non‐tumour tissues (n = 34). C, No significant correlations were observed between SCD gene expression levels and tumour T stage. D, No significant correlations were observed between SCD gene expression levels and tumour pathological stage. E, Negative IHC staining of SCD was observed in non‐tumour tissues; positive IHC staining of SCD was observed in tumour tissues. F, Western blot showed that SCD protein was overexpressed in cancer tissues compared to non‐tumour tissues. G, Western blot showed that SCD protein was overexpressed in all six bladder cancer cell lines. H, qRT‐PCR showed that high level of SCD mRNA was observed in all six bladder cancer cell lines

We then analysed the clinical tissue specimens collected from our hospital to verify the results of the bioinformatics analyses. We confirmed that SCD gene expression was significantly up‐regulated in tumour tissues compared to that in normal bladder mucosal tissues (Figure 3A,B); no significant correlations were observed between SCD gene expression and tumour stage (Figure 3C,D). To examine the expression of SCD in clinical bladder cancer specimens, four pairs of bladder cancer tissues and adjacent non‐tumour bladder tissues were tested. The results of immunohistochemistry and western blotting staining all suggested a significant elevation of SCD gene expression in tumour tissues compared to that in adjacent normal bladder mucosal tissues (Figure 3E,F). Western blot and qRT‐PCR were also performed on six cultured bladder cancer cell lines, and a similar result was observed (Figure 3G,H). Taken together, these data suggest that SCD is highly expressed in bladder tumours and could be a reliable target for further investigations.

### 3.3 Blockade of SCD activity inhibits the proliferation of bladder cancer cell lines

**Figure 4:**
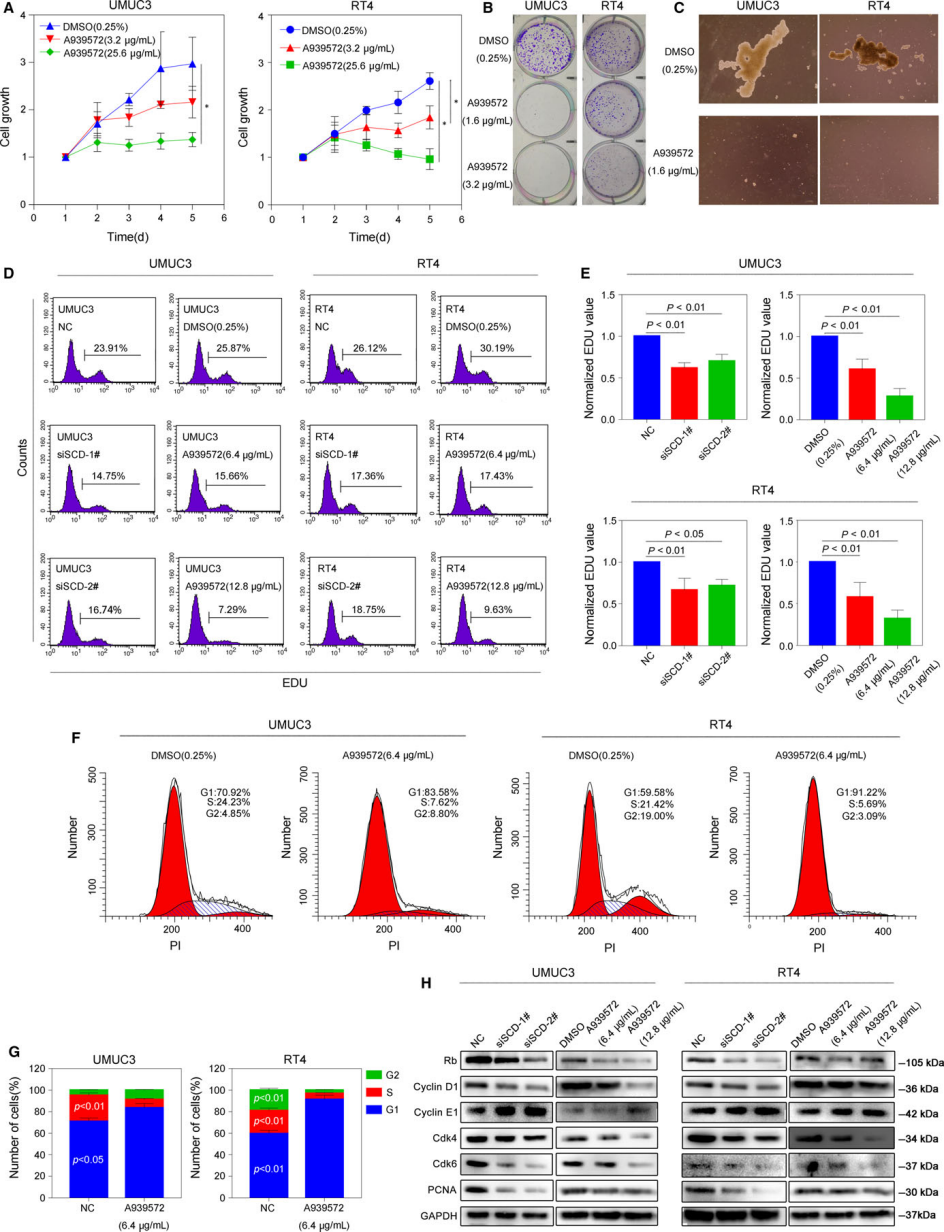
Blockade of SCD activity inhibits the proliferation of bladder cancer cell lines. A, The inhibition of SCD activity (using A939572 at the concentration of 3.2 or 25.6 µg/mL) significantly decreased the growth of UMUC3 (left) and RT4 (right) cells compared to that of the DMSO (0.25%) control, *P < 0.05. B, The colony formation assay revealed that after treatment of A939572 (1.6 or 3.2 µg/mL), UMUC3 (left) and RT4 (right) cells significantly lost their clonal ability. C, After 2 weeks of treatment of A939572 (1.6 µg/mL), UMUC3 (left) and RT4 (right) cells lost their sphere formation ability. D and E, Compared with the DMSO‐treated group or the normal control, SCD inhibition using either A939572 or two siRNAs significantly decreased cell proliferation. F and G, After treatment of A939572 (6.4 µg/mL), cell cycle was significantly arrested in the G1 phase. H, after either 24‐h treatment of A939572 or siRNAs, Cyclins D1, Rb, PCNA, Cdk4 and Cdk6 were down‐regulated, while cyclin E1 was up‐regulated

To examine whether the SCD gene could affect the cellular function of bladder cancer cells, we inhibited SCD activity using two SCD‐specific siRNAs and the SCD‐specific inhibitor A939572. Two cell lines, UMUC3, a highly malignant cell line, and RT4, a transitional papilloma cell line, were used to perform a proliferation assay. As shown in Figure 4A, the growth curve revealed that after treatment with 3.2 or 25.6 µg/mL A939572, the inhibition of SCD activity significantly decreased the growth of UMUC3 and RT4 cells compared to that of the DMSO (0.25%) control. The colony formation assay revealed similar results, and the concentrations used for SCD blockade were 1.6 and 3.2 µg/mL (Figure 4B). As we identified SCD as a target using a BCSC microarray screening assay, a sphere formation assay was then performed to confirm the influence of SCD during the course of CSC formation. After 2 weeks of treatment at a concentration of 1.6 µg/mL A939572, we observed that UMUC3 and RT4 cells notably lost their sphere formation ability (Figure 4C). To obtain stronger evidence, we constructed SCD‐knockdown cell lines using two SCD‐specific siRNAs combined with an inhibitor‐treated group. We next performed an EdU assay to determine the effect of SCD on cell proliferation using flow cytometry. As shown in Figure 4D,E, compared with the DMSO‐treated group or the normal control, SCD inhibition using either A939572 or two siRNAs significantly decreased the EdU values. A cell cycle assay was also performed, and we found that the cell cycle was significantly arrested in the G1 phase. Compared to that of the DMSO group, the number of cells in the G1 phase increased by over 10% in the inhibitor‐treated group (Figure 4F,G). Accordingly, several common cell cycle‐related proteins were detected in each group by western blotting. Cyclins D1, Rb, Cdk4 and Cdk6 were down‐regulated, while cyclin E1 was up‐regulated. Not surprisingly, PCNA was down‐regulated (Figure 4H). All data indicated that SCD directly regulates bladder cancer cell growth. Blockade of SCD activity dramatically inhibited cell proliferation and caused cell cycle arrest in the G1/S phase.

### 3.4 Inhibition of SCD blocks the migration and invasion of bladder cancer cell lines in vitro

**Figure 5:**
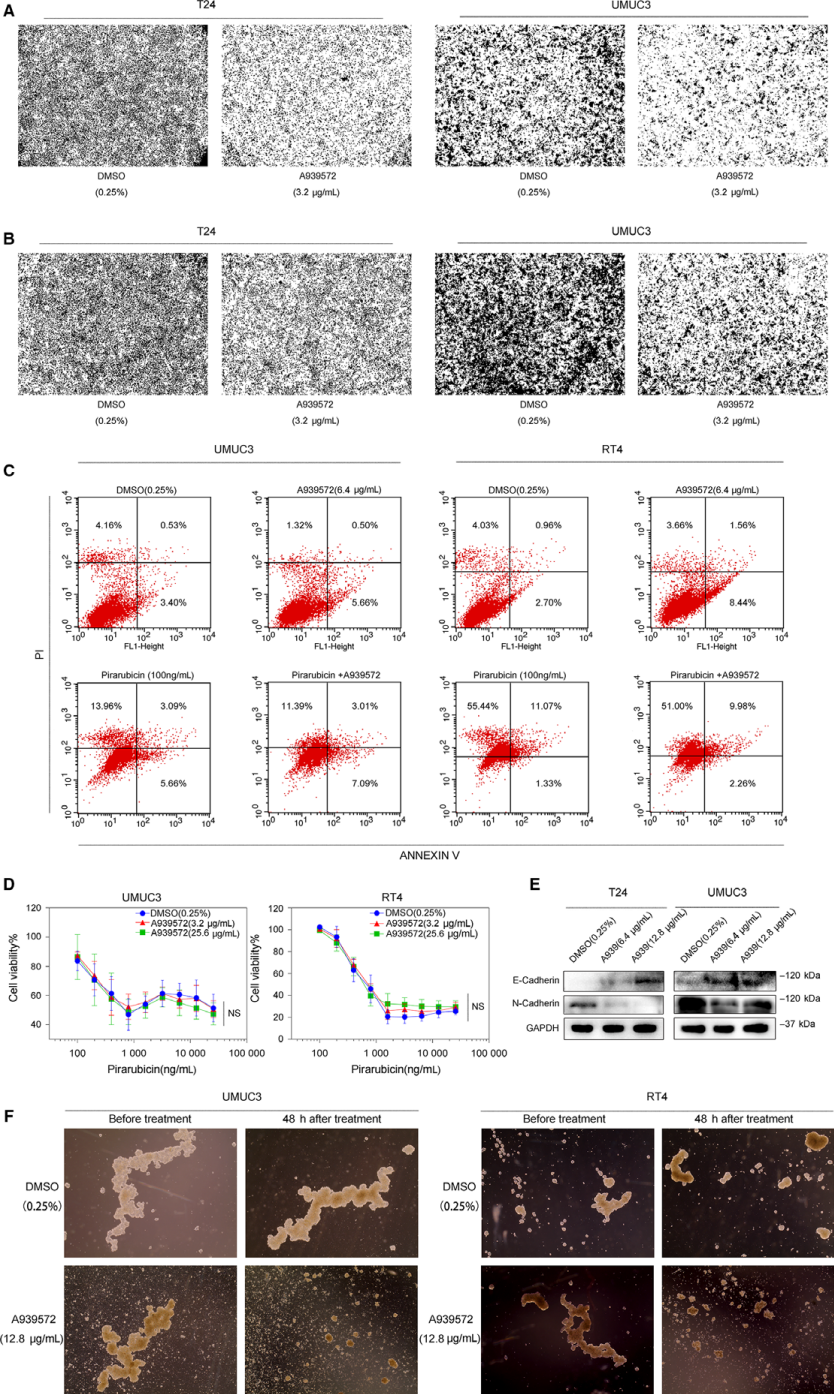
Inhibition of SCD blocks the migration and invasion of bladder cancer cell line; the combination of an SCD inhibitor and pirarubicin does not enhance cancer apoptosis. A, After treatment of A939572 (3.2 µg/mL), the invasion ability of T24 and UMUC3 cell lines were significantly decreased. B, After treatment of A939572 (3.2 µg/mL), the migration ability of T24 and UMUC3 cell lines were significantly decreased. C, Compared with treatment with DMSO, treatment with pirarubicin alone (100 ng/mL) significantly increased cell apoptosis. However, the combination of pirarubicin with A939572 (6.4 µg/mL) did not improve the effect of the drug. A939572 treatment alone had no effect on cell apoptosis. D, Combined with A939572 (3.2 and 25.6 µg/mL), the inhibitory effect of pirarubicin in UMUC3 (left) or RT4 (right) cells did not increase. E, Compared to the DMSO (0.25%) group, A939572 at concentrations of 6.4 and 12.8 µg/mL up‐regulated E‐cadherin expression and down‐regulated N‐cadherin expression. F, Compared to the control group, the outline of the spheres in the treatment group (A939572 alone at a concentration of 12.8 µg/mL for 48 h) was significantly smaller

To further explore the effect of the SCD gene on cellular functions, in vitro cell invasion and migration assays were performed. We used two aggressive bladder cancer cell lines, T24 and UMUC3, for the test. As shown in Figure 5A,B, when the inhibitor was added at a concentration of 3.2 µg/mL, the invasion and migration abilities of the T24 and UMUC3 cell lines were significantly decreased. E‐cadherin and N‐cadherin, the most common epithelial‐mesenchymal transition markers, are considered to be markers of migration and invasion. The cells were pre‐treated with A939572 at concentrations of 6.4 and 12.8 µg/mL. Compared to that in the DMSO (0.25%) group, N‐cadherin expression was remarkably down‐regulated in inhibitor‐treated cell lines, whereas E‐cadherin expression was up‐regulated (Figure 5E). In summary, SCD inhibition was capable of decreasing the migration and invasion abilities of bladder cancer cell lines in vitro.

### 3.5 The combination of an SCD inhibitor and pirarubicin does not enhance cancer apoptosis in vitro

Many studies reported that pharmacologic or genetic interference with SCD remarkably accelerated the process of cellular apoptosis in different types of cancer. Moreover, SCD inhibition could also significantly increase the sensitivity of cells to chemotherapy‐induced apoptosis.^12^ To determine whether a similar result could also be observed in bladder cancer cells after treatment with A939572 and pirarubicin alone or in combination, we performed an apoptosis assay in both the UMUC3 and RT4 cell lines. The results suggested that compared with treatment with DMSO, treatment with pirarubicin alone at a concentration of 100 ng/mL significantly increased cell apoptosis. However, the combination of pirarubicin with A939572 at a concentration of 6.4 µg/mL did not improve the effect of the drug. In addition, A939572 treatment alone had no effect on cell survival (Figure 5C). The cell viability assay showed a similar trend. When combined with A939572 at concentrations of 3.2 and 25.6 µg/mL, the inhibitory effect of pirarubicin in UMUC3 or RT4 cells did not increase (Figure 5D). Interestingly, in CSCs, we observed a different result. As shown in Figure 5F, we treated UMUC3 and RT4 spheres with A939572 alone at a concentration of 12.8 µg/mL for 48 hours. Compared to that of the control group, the outline of the spheres in the treatment group was significantly smaller. Accordingly, the number of cell fragments in the background also remarkably increased. Taken together, the above data suggested that the SCD inhibitor was unable to reduce cell survival in 2D cultured cells, but in bladder CSCs, cell apoptosis was enhanced after treatment with A939572.

## 4 DISCUSSION

To date, there have been few SCD studies in the field of bladder urothelial cancer. In our study, we elaborated for the first time on the relationship between SCD gene activity and bladder cancer. More importantly, we generated BCSCs from human bladder cancer cell lines and performed a microarray assay to further identify DEGs. With the results of the two datasets combined, using Lee bladder data from ONCOMINE and TCGA, we selected SCD as the most crucial target among the 17 identified DEGs. Next, to verify this result, we tested the mRNA level of SCD in 25 paired and 91 unpaired normal bladder mucosal and/or tumour tissue specimens by real‐time PCR. Not surprisingly, compared with that of the normal tissues, the SCD mRNA level was significantly elevated in tumours. The results of western blotting and immunohistochemistry revealed the same tendency. In vitro experiments suggested that SCD strongly promoted tumour cell proliferation and invasion. Finally, we tested cell inhibition after combined treatment with an SCD inhibitor and pirarubicin. No positive results were obtained. Thus, we conclude that high SCD expression indicates poor prognosis in patients with bladder urothelial cancer, and the blockade of SCD gene activity significantly inhibits the proliferation and invasion of bladder cancer cells. Unlike many other types of cancer, the inhibition of SCD may not increase the efficacy of conventional chemotherapy in bladder cancer. However, SCD inhibitors may target CSCs specifically to suppress proliferation and evoke apoptosis in CSCs in patients with bladder cancer.

There is growing evidence of unbalanced levels of SFAs and MUFAs in the blood and tissues of cancer patients, which may indicate abnormal SCD activity in the disease. Many clinical studies have shown that MUFA is maintained at a high level in the plasma and tissues of cancer patients. This phenomenon was related not only to the presence of cancer, but also to poor patient prognosis and higher cancer mortality.^13^, ^14^, ^15^, ^16^, ^17^ For a long time, abnormal fatty acid composition has been found in in vitro and in vivo experiments. It is characterized by high MUFA and decreased levels of SFA and polyunsaturated fatty acid.^18^, ^19^, ^20^, ^21^, ^22^, ^23^, ^24^, ^25^

By performing gene‐level studies in tumour cells, we identified the central role of SCD in the mechanisms of cell replication. With the use of a small molecule SCD inhibitor,^26^ which could be selectively against Δ5 and Δ6 desaturases, or gene interference, studies have shown that the proliferation of cancer cells strictly depends on SCD activity.^27^, ^28^, ^29^ These experiments have, for the first time, found direct evidence that SCD is a key factor of tumour cell proliferation and survival. Subsequently, a series of researches showed that SCD inhibition has anti‐proliferative and apoptosis effects in a variety of neoplasm cells.^30^, ^31^, ^32^, ^33^, ^34^, ^35^, ^36^, ^37^, ^38^, ^39^, ^40^

More evidence suggests that SCD modulates the replication rate of tumour cells by regulating certain factors in cell cycle progression. Studies performed in lung cancer cells found that when SCD activity was inhibited, cancer cells were arrested in the G1 phase of the cell cycle.^41^ The researchers demonstrated that the activity of relevant regulatory factors is dependent on the synthesis of MUFA.^41^ One study showed that SCD, to some extent, controls cell cycle progression by regulating the expression levels of cyclin D1 and CDK6.^42^ It was also reported that the inhibition of SCD in cancer cells could reduce the expression level of nuclear β‐catenin, which can activate the expression of cyclin D1.^38^ However, studies related to the changes of MUFA synthesis and the activation and progression of the cell cycle have yet to be elucidated.

Elegant studies clearly demonstrated that SCD activity is necessary for the replication and differentiation of human pluripotent stem cells. However, once the cells have completed the differentiation process, the desaturase activity is no longer the key for cell survival.^43^, ^44^

Stearoyl CoA desaturase‐1 has been shown to control many of the biological properties of cancer cells. Therefore, many studies reported that high levels of MUFA synthesis are essential for cancer development and growth. It is worth noting that when a glycolytic cell line MIA Paca‐2, and a lipogenic cell line HPAC that expresses high levels of SCD and MUFAs, were treated with A37062, a SCD small molecule inhibitor, remarkable tumour growth suppression was observed only in the HPAC‐derived xenografted tumour.^45^ Although the antitumour effect of SCD suppression has been reported, milder therapeutic effect was observed in some cancers. In xenograft tumours deriving from MF‐438 cells, treatment with the SCD inhibitor A939572 had no virtual antitumour impacts but suppressed tumour growth when carfilzomib was used.^37^ In our study, the results suggested that treatment with A939572 did not cause apoptosis in parental bladder cancer cell lines. However, in CSCs, using an SCD inhibitor alone may reduce CSC proliferation and increase apoptosis.

However, the present study has several limitations. First, all confirmatory experiments were performed in vitro. In vivo data are necessary to support our findings, and in vivo experiments are our next step in confirming our results. Next, we were not able to explore the exact mechanism that underlies the biological function of SCD in bladder carcinoma. SCD regulates important survival and proliferation signalling pathways in tumour cells. The levels of SCD activity affect the carcinogenic phenotype by modulating critical oncogenic signalling pathways, especially AMPK, PI3K‐Akt, Wnt, mTOR and NF‐kB pathways.^46^, ^47^, ^48^, ^49^, ^50^ To explore these mechanisms more fully, lipid metabolomic analysis and transcriptome analysis are necessary. Finally, the results of the present study may be a chance finding due to the limited sample size and the lack of additional validation. Therefore, additional large‐scale and independent studies are essential.

## CONFLICT OF INTEREST

The authors confirm that there are no conflicts of interest.

## References

[1] Miller KD , Siegel RL , Lin CC , et al. Cancer treatment and survivorship statistics, 2016. CA Cancer J Clin. 2016;66:271‐289. 27253694 10.3322/caac.21349

[2] Chen W , Zheng R , Baade PD , et al. Cancer statistics in China, 2015. CA Cancer J Clin. 2016;66:115‐132. 26808342 10.3322/caac.21338

[3] Antoni S , Ferlay J , Soerjomataram I , et al. Bladder cancer incidence and mortality: a global overview and recent trends. Eur Urol. 2017;71:96‐108. 27370177 10.1016/j.eururo.2016.06.010

[4] Millan‐Rodriguez F , Chechile‐Toniolo G , Salvador‐Bayarri J , et al. Multivariate analysis of the prognostic factors of primary superficial bladder cancer. J Urol. 2000;163:73‐78. 10604317 10.1016/s0022-5347(05)67975-x

[5] Fialkow PJ , Jacobson RJ , Papayannopoulou T . Chronic myelocytic leukemia: clonal origin in a stem cell common to the granulocyte, erythrocyte, platelet and monocyte/macrophage. Am J Med. 1977;63:125‐130. 267431 10.1016/0002-9343(77)90124-3

[6] Bonnet D , Dick JE . Human acute myeloid leukemia is organized as a hierarchy that originates from a primitive hematopoietic cell. Nat Med. 1997;3:730‐737. 9212098 10.1038/nm0797-730

[7] Clarke MF , Dick JE , Dirks PB , et al. Cancer stem cells–perspectives on current status and future directions: AACR Workshop on cancer stem cells. Cancer Res. 2006;66:9339‐9344. 16990346 10.1158/0008-5472.CAN-06-3126

[8] Kurzrock EA , Lieu DK , Degraffenried LA , et al. Label‐retaining cells of the bladder: candidate urothelial stem cells. Am J Physiol Renal Physiol. 2008;294:F1415‐F1421. 18367656 10.1152/ajprenal.00533.2007

[9] Chan KS , Espinosa I , Chao M , et al. Identification, molecular characterization, clinical prognosis, and therapeutic targeting of human bladder tumor‐initiating cells. Proc Natl Acad Sci USA. 2009;106:14016‐14021. 19666525 10.1073/pnas.0906549106 PMC2720852

[10] Goodwin Jinesh G , Willis DL , Kamat AM . Bladder cancer stem cells: biological and therapeutic perspectives. Curr Stem Cell Res Ther. 2014;9:89‐101. 24236543 10.2174/1574888x08666131113123051

[11] Tran MN , Goodwin Jinesh G , McConkey DJ , et al. Bladder cancer stem cells. Curr Stem Cell Res Ther. 2010;5:387‐395. 20955163 10.2174/157488810793351640

[12] Pisanu ME , Noto A , De Vitis C , et al. Blockade of stearoyl‐CoA‐desaturase 1 activity reverts resistance to cisplatin in lung cancer stem cells. Cancer Lett. 2017;406:93‐104. 28797843 10.1016/j.canlet.2017.07.027

[13] Bougnoux P , Chajes V , Lanson M , et al. Prognostic significance of tumor phosphatidylcholine stearic acid level in breast carcinoma. Breast Cancer Res Treat. 1992;20:185‐194. 1571571 10.1007/BF01834624

[14] Zhu ZR , Agren J , Mannisto S , et al. Fatty acid composition of breast adipose tissue in breast cancer patients and in patients with benign breast disease. Nutr Cancer. 1995;24:151‐160. 8584451 10.1080/01635589509514403

[15] Zureik M , Ducimetiere P , Warnet JM , et al. Fatty acid proportions in cholesterol esters and risk of premature death from cancer in middle aged French men. BMJ. 1995;311:1251‐1254. 7496232 10.1136/bmj.311.7015.1251 PMC2551179

[16] Simonsen NR , Fernandez‐Crehuet Navajas J , Martin‐Moreno JM , et al. Tissue stores of individual monounsaturated fatty acids and breast cancer: the EURAMIC study. European Community Multicenter Study on Antioxidants, Myocardial Infarction, and Breast Cancer. Am J Clin Nutr. 1998;68:134‐141. 9665107 10.1093/ajcn/68.1.134

[17] Chajes V , Joulin V , Clavel‐Chapelon F . The fatty acid desaturation index of blood lipids, as a biomarker of hepatic stearoyl‐CoA desaturase expression, is a predictive factor of breast cancer risk. Curr Opin Lipidol. 2011;22:6‐10. 20935562 10.1097/MOL.0b013e3283404552

[18] Haven FL , Bloor WR , Randall C . The nature of the fatty acids of rats growing Walker carcinoma 256. Cancer Res. 1951;11:619‐623. 14859226

[19] Medes G , Thomas A , Weinhouse S . Metabolism of neoplastic tissue. IV. A study of lipid synthesis in neoplastic tissue slices in vitro. Cancer Res. 1953;13:27‐29. 13032945

[20] Stein AA , Opalka E , Rosenblum I . Fatty acid analysis of two experimental transmissible glial tumors by gas‐liquid chromatography. Cancer Res. 1965;25:201‐205. 14264052

[21] Yau TM , Weber MJ . Changes in acyl group composition of phospholipids from chicken embryonic fibroblasts after transformation by Rous sarcoma virus. Biochem Biophys Res Commun. 1972;49:114‐120. 4342719 10.1016/0006-291x(72)90016-2

[22] Hale AH , Yau TM , Weber MJ . Membrane lipid acyl group alterations in cells infected with a temperature‐conditional mutant of Rous sarcoma virus. Biochim Biophys Acta. 1976;443:618‐622. 183825 10.1016/0005-2736(76)90481-8

[23] Fallani A , Ruggieri S . Lipid composition of SV40‐induced transplantable hamster tumor. Lipids. 1979;14:752‐755. 231174 10.1007/BF02533902

[24] Peel WE , Thomson AE . The fatty acyl chain composition of human normal and leukaemic lymphocytes and its modulation by specialised hydrogenation. Leuk Res. 1983;7:193‐204. 6855267 10.1016/0145-2126(83)90009-7

[25] Zoeller RA , Wood R . The importance of the stearoyl‐CoA desaturase system in octadecenoate metabolism in the Morris hepatoma 7288C. Biochim Biophys Acta. 1985;845:380‐388. 2860925 10.1016/0167-4889(85)90202-2

[26] Koltun DO , Parkhill EQ , Vasilevich NI , et al. Novel, potent, selective, and metabolically stable stearoyl‐CoA desaturase (SCD) inhibitors. Bioorg Med Chem Lett. 2009;19:2048‐2052. 19249203 10.1016/j.bmcl.2009.02.019

[27] Scaglia N , Chisholm JW , Igal RA . Inhibition of stearoylCoA desaturase‐1 inactivates acetyl‐CoA carboxylase and impairs proliferation in cancer cells: role of AMPK. PLoS ONE. 2009;4:e6812. 19710915 10.1371/journal.pone.0006812 PMC2728543

[28] Scaglia N , Igal RA . Stearoyl‐CoA desaturase is involved in the control of proliferation, anchorage‐independent growth, and survival in human transformed cells. J Biol Chem. 2005;280:25339‐25349. 15851470 10.1074/jbc.M501159200

[29] Scaglia N , Igal RA . Inhibition of stearoyl‐CoA desaturase 1 expression in human lung adenocarcinoma cells impairs tumorigenesis. Int J Oncol. 2008;33:839‐850. 18813799

[30] Nashed M , Chisholm JW , Igal RA . Stearoyl‐CoA desaturase activity modulates the activation of epidermal growth factor receptor in human lung cancer cells. Exp Biol Med (Maywood). 2012;237:1007‐1017. 22946088 10.1258/ebm.2012.012126

[31] Du X , Wang QR , Chan E , et al. FGFR3 stimulates stearoyl CoA desaturase 1 activity to promote bladder tumor growth. Cancer Res. 2012;72:5843‐5855. 23019225 10.1158/0008-5472.CAN-12-1329

[32] Fritz V , Benfodda Z , Rodier G , et al. Abrogation of de novo lipogenesis by stearoyl‐CoA desaturase 1 inhibition interferes with oncogenic signaling and blocks prostate cancer progression in mice. Mol Cancer Ther. 2010;9:1740‐1754. 20530718 10.1158/1535-7163.MCT-09-1064 PMC3315476

[33] Roongta UV , Pabalan JG , Wang X , et al. Cancer cell dependence on unsaturated fatty acids implicates stearoyl‐CoA desaturase as a target for cancer therapy. Mol Cancer Res. 2011;9:1551‐1561. 21954435 10.1158/1541-7786.MCR-11-0126

[34] Peck B , Schug ZT , Zhang Q , et al. Inhibition of fatty acid desaturation is detrimental to cancer cell survival in metabolically compromised environments. Cancer Metab. 2016;4:6. 27042297 10.1186/s40170-016-0146-8 PMC4818530

[35] Kim SJ , Choi H , Park SS , et al. Stearoyl CoA desaturase (SCD) facilitates proliferation of prostate cancer cells through enhancement of androgen receptor transactivation. Mol Cells. 2011;31:371‐377. 21331774 10.1007/s10059-011-0043-5 PMC3933960

[36] von Roemeling CA , Marlow LA , Wei JJ , et al. Stearoyl‐CoA desaturase 1 is a novel molecular therapeutic target for clear cell renal cell carcinoma. Clin Cancer Res. 2013;19:2368‐2380. 23633458 10.1158/1078-0432.CCR-12-3249 PMC3644999

[37] von Roemeling CA , Marlow LA , Pinkerton AB , et al. Aberrant lipid metabolism in anaplastic thyroid carcinoma reveals stearoyl CoA desaturase 1 as a novel therapeutic target. J Clin Endocrinol Metab. 2015;100:E697‐709. 25675381 10.1210/jc.2014-2764 PMC4422887

[38] Mauvoisin D , Charfi C , Lounis AM , et al. Decreasing stearoyl‐CoA desaturase‐1 expression inhibits beta‐catenin signaling in breast cancer cells. Cancer Sci. 2013;104:36‐42. 23013158 10.1111/cas.12032 PMC7657162

[39] Zhang Y , Wang H , Zhang J , et al. Positive feedback loop and synergistic effects between hypoxia‐inducible factor‐2alpha and stearoyl‐CoA desaturase‐1 promote tumorigenesis in clear cell renal cell carcinoma. Cancer Sci. 2013;104:416‐422. 23331615 10.1111/cas.12108 PMC7657137

[40] Noto A , Raffa S , De Vitis C , et al. Stearoyl‐CoA desaturase‐1 is a key factor for lung cancer‐initiating cells. Cell Death Dis. 2013;4:e947. 24309934 10.1038/cddis.2013.444 PMC3877537

[41] Hess D , Chisholm JW , Igal RA . Inhibition of stearoylCoA desaturase activity blocks cell cycle progression and induces programmed cell death in lung cancer cells. PLoS ONE. 2010;5:e11394. 20613975 10.1371/journal.pone.0011394 PMC2894866

[42] Malumbres M , Barbacid M . Cell cycle, CDKs and cancer: a changing paradigm. Nat Rev Cancer. 2009;9:153‐166. 19238148 10.1038/nrc2602

[43] Ben‐David U , Gan QF , Golan‐Lev T , et al. Selective elimination of human pluripotent stem cells by an oleate synthesis inhibitor discovered in a high‐throughput screen. Cell Stem Cell. 2013;12:167‐179. 23318055 10.1016/j.stem.2012.11.015

[44] Ben‐David U , Biran A , Scaffidi P , et al. Elimination of undifferentiated cancer cells by pluripotent stem cell inhibitors. J Mol Cell Biol. 2014;6:267‐269. 24683201 10.1093/jmcb/mju012 PMC4055828

[45] Daemen A , Peterson D , Sahu N , et al. Metabolite profiling stratifies pancreatic ductal adenocarcinomas into subtypes with distinct sensitivities to metabolic inhibitors. Proc Natl Acad Sci USA. 2015;112:E4410‐E4417. 26216984 10.1073/pnas.1501605112 PMC4538616

[46] Vivanco I , Sawyers CL . The phosphatidylinositol 3‐kinase AKT pathway in human cancer. Nat Rev Cancer. 2002;2:489‐501. 12094235 10.1038/nrc839

[47] Polakis P . The many ways of Wnt in cancer. Curr Opin Genet Dev. 2007;17:45‐51. 17208432 10.1016/j.gde.2006.12.007

[48] Hardie DG . Molecular pathways: is AMPK a friend or a foe in cancer? Clin Cancer Res. 2015;21:3836‐3840. 26152739 10.1158/1078-0432.CCR-14-3300 PMC4558946

[49] Zoncu R , Efeyan A , Sabatini DM . mTOR: from growth signal integration to cancer, diabetes and ageing. Nat Rev Mol Cell Biol. 2011;12:21‐35. 21157483 10.1038/nrm3025 PMC3390257

[50] Li J , Condello S , Thomes‐Pepin J , et al. Lipid desaturation is a metabolic marker and therapeutic target of ovarian cancer stem cells. Cell Stem Cell. 2017;20:303‐14.e5. 28041894 10.1016/j.stem.2016.11.004 PMC5337165

